# Effects of Dietary Inclusion of *Spirulina platensis* on the Reproductive Performance of Female Mink

**DOI:** 10.3390/vetsci9080428

**Published:** 2022-08-12

**Authors:** Anna Maria Iatrou, Georgios A. Papadopoulos, Ilias Giannenas, Aristotelis Lymberopoulos, Paschalis Fortomaris

**Affiliations:** 1 Laboratory of Animal Husbandry, School of Veterinary Medicine, Faculty of Health Sciences, Aristotle University of Thessaloniki, 54124 Thessaloniki, Greece; 2 Laboratory of Nutrition, School of Veterinary Medicine, Faculty of Health Sciences, Aristotle University of Thessaloniki, 54124 Thessaloniki, Greece; 3 Laboratory of Farm Animal Reproduction and Breeding, Division of Animal Science, Department of Agriculture, School of Geosciences, International Hellenic University, 57400 Thessaloniki, Greece

**Keywords:** mink, Spirulina, reproduction, whelping rate, body weight

## Abstract

**Simple Summary:**

Profitability of mink farming depends mainly on the reproductive performance of female animals. Many dietary practices applied previously, to improve health and productivity of mink. *Spirulina platensis* is a natural dietary supplement, widely used both for human and animal consumption. In previous studies, in many animal species, *Spirulina platensis* supplementation improved reproductive characteristics. To our knowledge, inclusion of Spirulina in a mink’s diet has not yet been adequately studied. To address this dearth of knowledge, we investigated the effect of dietary inclusion of *Spirulina platensis* on the reproductive performance of female mink from pre-mating season until kit weaning. A tendency for a higher whelping rate was detected in *Spirulina platensis* treated animals, while a significant increase in their body weight during pre-mating season was observed. Finally, *Spirulina platensis* treated animals lost significantly more body weight during late lactation, but without an adverse effect on their or their kit’s survival. Overall, it can be suggested that *Spirulina platensis* can be used in female mink diets without adverse effects. However, since in the present study only a single dose was used, further studies are required to investigate the effects of higher levels of supplementation.

**Abstract:**

The objective of this study was to investigate the impact of providing *Spirulina platensis* (Spirulina) on reproductive performance of female mink. A total of 100 adult brown female mink (*Mustela vison*) were randomly and equally allocated to control group (C group), in which mink were fed basal diet and Spirulina group (Sp group), where mink received basal diet supplemented with 100 mg of Spirulina/kg of body weight. The experiment lasted 5 months, starting from 1 month prior to mating till kit weaning. Weight gain during pre-mating period was higher in Sp group compared to C group (*p* < 0.001). Sp group remained heavier until the onset of lactation. Subsequently, mink of Sp group lost more weight than C group (*p* < 0.001) but without an adverse effect on kit survival. A tendency for a higher whelping rate was detected in Sp group (93.61%) compared to C group (81.25%) (*p* = 0.07). Litter size, as well as weight of kits at weaning, did not differ between groups (*p* > 0.10). Finally, Sp group weaned numerically more kits compared to C group. Results obtained here showed that Spirulina treated animals tended to an increased whelping rate.

## 1. Introduction

Mink (*Mustela vison*) are carnivorous animals, bred worldwide for their fur. Profitability of mink industry is based on the reproductive performance of female breeders as well as on the amount and size of the pelt [1,2,3]. In common breeding programs of mink farming, the selection of female breeders mainly depends on their increased litter size and body weight, phenotypical traits characterized by a negative correlation [2,4].

Feed is the major production cost in a mink farm, and typically is based on meat and fish by-products [2,5]. The peri-parturient period, as well as weaning of kits, is a very stressful period for mink dams, due to metabolic, nutritional, physical, or environmental factors, which leads to reduction in body weight and animal losses. This condition has been described in mink as a nursing disease [6]. The nutritional and especially energy demands of mink change throughout the year and are notably high during the mating, gestation, and lactation periods [5,7,8]. The required energy is mainly supplied by proteins and fat [5]. In general, mink diets contain more fat compared to other domestic animals [9]. Sources of fat are usually fish and fish by-products, which are characterized by a moderate level of polyunsaturated fatty acids (PUFA) and by a higher level of monounsaturated fatty acids (MUFA) [10]. Additionally, fetal protein plasma concentrations, growth, and development of fetus are strongly connected to maternal dietary protein status [11]. During such periods of intensive feeding, which may lead to an increased metabolic rate, an increased production of Reactive Oxygen Species (ROS) may occur [12]. Oxidative stress and ROS could cause cell damage and inhibit DNA normal function [9]. Thus, oxidative stress may affect not only the fertility of mink dams but also the well-being of mink offspring. In this context, previous studies indicated that dietary inclusion of antioxidants, e.g., vitamin E and selenium, in mink diets could prevent and delay oxidative processes [9].

*Spirulina platensis* (Spirulina), is a dietary supplement with high nutritional value, produced by cyanobacteria [13,14]. Spirulina contains approximately 50–70% proteins, all essential amino acids, vitamins, and mineral elements, such as Zn, Mg, and Se, while it is also rich in bioactive compounds, such as phycocyanin, tocopherols, phenolics, carotenoids, and fatty acids, especially γ-linolenic acid [14,15,16,17,18]. It should also be noted that Spirulina has been used as an alternative natural carotenoid source in various fish species’ diets [19,20]. Due to its content in antioxidative vitamins and carotenoids, positive effects on fertility in several species, including mink, were reported [1,9].

To our knowledge, inclusion of Spirulina in a mink’s diet has not yet been extensively studied. Previously, supplementation with Spirulina in a mink’s diet improved their reproductive performance, by reducing the percentage of abortive females and, at the same time, increasing litter size of females at birth [21]. Nevertheless, in the latter study, no data during the whole reproductive period were shown. Based on its described properties, we hypothesized that dietary supplementation of Spirulina compared to un-supplemented commercial diets fed to female mink during the complete reproductive period could be beneficial for their reproductive performance.

The objective of the present study was to assess the impact of Spirulina supplementation on the reproductive performance of female minks during periods of high metabolic demand, such as mating, gestation, and lactation.

## 2. Materials and Methods

### 2.1. Animals

**Figure 1:**
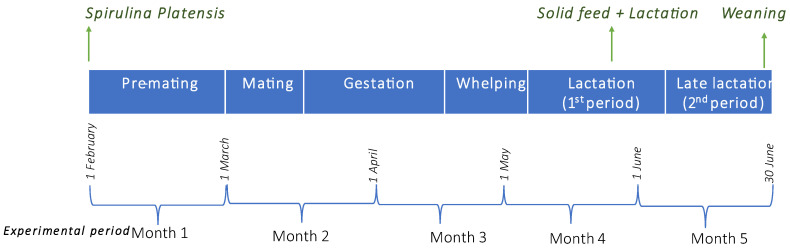
Experimental period layout. The experiment lasted since 1 February (one month prior to mating season) till end of June. Mating season started on 1 March and lasted till 15th of the same month. Whelping period lasted from 20 April till 5 May. From 24 May kits had access to solid feed and lactation. Weaning of kits was on 28 June, and after that, offspring were separated from their dams.

The experiment was part of the Ph.D. thesis of A.M.I. and was approved by the General Assembly of the School of Veterinary Medicine, Faculty of Health Science, Aristotle University of Thessaloniki (decision 627/21-1-2020). The study was carried out in a commercial mink farm in Kozani prefecture, in Northern Greece. In total, 100 healthy, free of Aleutian Disease Virus, adult, brown female mink (*Mustela vison*) were randomly selected and enrolled in the study. Animals were kept under conventional mink farm conditions and were fed a standard mink diet. Mink were housed in roofed sheds with open sides, in individual standard mink cages (30 width × 92 length × 46 height cm) with attached wooden nest boxes and bedded with straw. Experimental period lasted five months (1st of February to end of June) (Figure 1).

### 2.2. Diet

Animals were randomly and equally allocated to a control group (C group, *n* = 50), where minks were fed the basal diet and a Spirulina group (Sp group, *n* = 50), where the basal diet was supplemented with 100 mg of Spirulina (powder) per kg of body weight. Spirulina was supplied from Hellenic Spirulina Net, and supplementation started 1 month prior to mating season and lasted until kit weaning. Dosage was monthly, calculated according to the average dams’ body weight. Spirulina powder was firstly dissolved in 500 mL of fresh water and then was mixed with the basal feed for mink. Animals had free access to drinking water from a nipple drinker throughout the experimental period. Weather conditions and body score of animals were monitored for the feed intake during the pre-mating and mating season, as also at gestation until whelping. Four weeks post-partum kits started to have access to solid feed additionally to milk and were fed the same feed as their dams, until weaning.

### 2.3. Feed Analysis

#### 2.3.1. Chemical Analysis of Feed

Feed samples were collected every 15 days and first sample was taken at the end of mating period. Control feed samples were collected directly from feed silo and Spirulina feed samples were collected directly from the feed machine, after the preparation of the mixture. Samples were kept in the refrigerator of the mink farm, were transported to the laboratory frozen, and were kept in the refrigerator of the laboratory until their analysis.

Six samples for each group (diet) were analyzed and mean values are provided. Chemical analysis of feed samples was carried out after drying, by Perten Instruments DA7250. A chemical analysis of Spirulina powder was also performed.

#### 2.3.2. Diet Fatty Acid Profile

Fatty acid composition of the mink feed was determined by gas chromatography; fatty acids methyl esters were obtained from the frozen samples. Subsequently, separation and quantification of the methyl esters was carried out with a gas chromatographic system (Trace GC model K07332, Thermo Finnigan, Thermo Quest, Milan, Italy) equipped with a flame ionization detector, a 5.01 version of Chrom-Card data system (Thermo Electron Corporation, Milan, Italy) and a fused silica capillary column, 30 m × 0.25 mm i.d., coated with cyanopropyl polysiloxane (phase type SP-2380) with a film thickness of 0.20 μm (Supelco, Bellefonte, PA, USA). The chromatographic conditions were: carrier: N2, flow: 1 mL/min; oven: temperature 37 °C for 4 min, increase 4 °C/min to 250 °C for 5 min; inlet temperature: 250 °C; detector temperature: 260 °C; and injection: 2 μL, with split 1/50. Fatty acid methyl ester retention time and elusion order were identified using as reference standard the Supelco ’37 Component FAME Mix’ (Sigma-Aldrich, Darmstadt, Germany ). Fatty acids were quantified by peak area measurement and the results were expressed as percentage (%) of the total peak areas for all quantified acids [22]. Fatty acids were classified to saturated, polyunsaturated, and monounsaturated.

### 2.4. Body Weights

Initial body weights (BW) of dams were measured on 30 January and means of body weight were similar between groups. To study the effect of Spirulina on BW of dams, they were weighed monthly. Animals were not weighed on April to avoid stress in the whelping period. A second measurement (1 month) was conducted before the beginning of mating period at the end of February, after one month of administrating Spirulina. At late March, during gestation period, a third measurement (2 month) of 40 out of 50 initial allocated animals of each group, took place. Due to practical reasons, 8 animals of C group and 7 animals of Sp group were not weighed. A fourth measurement (4 month) was carried out at late May, during lactation period and a fifth measurement (5 month) was conducted at late June, at weaning stage. For the calculation of dosage of Spirulina during whelping and lactation period (May), five animals of each group were weighed after whelping, but they were not included in the statistical analysis. Kits were weighed once at the weaning, at the end of June.

### 2.5. Reproductive Performance

Mating started in early March and all the breeding females were yearlings. Animals mated according to the normal mating system 1-8-1 [23], meaning that they had one simple mating on the first date and a double mating, on two consecutive days, eight and nine days later, respectively.

Date of each mating, date of parturition, and litter size were recorded. During whelping period which lasted from 22 April till 5 May, all females were daily monitored for evidence of whelping. Kits were counted on the day of birth, 10 days post-partum (d10) and at weaning on 28 June. Pre-weaning mortality was calculated from birth till 28 June.

Values for the reproductive performance were analyzed: mated females (percentage of females that mated at least once), whelping rate (percentage of mated females that gave birth), barren females (females that mated at least once but there was no parturition), litter size at birth, day 10 (d10), and at weaning, mortality of kits (percentage of mortality of kits during maternal nursing).

Regarding pregnancy length, for dams mated only once, it was calculated as the days from mating to parturition. For the rest animals, second mating date was considered as the day of fertilization and the beginning of pregnancy.

Animals that did not mate and those that mated, but did not give birth, were removed from the experiment, at the end of mating and whelping period, respectively.

### 2.6. Statistical Analysis

Statistical analysis of most recorded parameters was performed using the SPSS v. 25.0 software (IBM Co., Chicago, IL, USA). Statistical analysis of mated females, whelped females and females that lost all their kits until weaning were conducted using a Chi-square independence test. For the values of litter size, mortality of kits, and weight of dams, as well as of kits at weaning a univariate analysis of variance (ANOVA) was used, with treatment group as a fixed factor. Weight differences of female mink between different stages during the reproductive period were analyzed with unpaired t-test in GraphPad Prism (version 9.1.2 for Windows^®^, GraphPad Software, San Diego, CA, USA). Values of *p* less than 0.05 were considered as statistically significant, whereas values between 0.05 < *p* < 0.10 were regarded as a “trend”.

## 3. Results

### 3.1. Feed Analysis

Chemical analysis, total phenolic content, and diet fatty acid profile of feed samples are presented in Table 1. Respectively, chemical analysis of Spirulina powder is presented in Table 2.

### 3.2. Reproduction Performance of Mink Dams and Mortality of Kits

Regarding C group, 48 out of 50 animals mated at least once and 39 of them gave birth (nine barren females). Respectively, 47 out of 50 animals from Sp group mated at least once and 44 of them whelped (three barren females). Compared to the C group, supplementation with Spirulina had no statistically significant difference regarding mating results (*p* > 0.05). A tendency for a higher whelping rate was detected in the animals supplemented with Spirulina (93.61%) compared to C group (81.25%) (*p* = 0.07). Six and five females from C and Sp group, respectively, lost all their kits until weaning and values were similar between two groups (*p* > 0.05). Litter size did not differ (*p* > 0.05) between groups which at birth, d10 and at weaning was 7.41, 5.41, and 5.02, and 6.95, 5.34, and 4.86 for C and Sp group, respectively. Mortality of kits was similar between groups (*p* > 0.05) (Table 3). Sp group weaned more kits (214) in comparison with C group (196) (*p* > 0.05). No difference was observed regarding average gestation length which was 47.13 days for C group and 45.22 days for Sp group (*p* > 0.05).

### 3.3. Weight of Female Mink and Kits at Weaning

**Figure 2:**
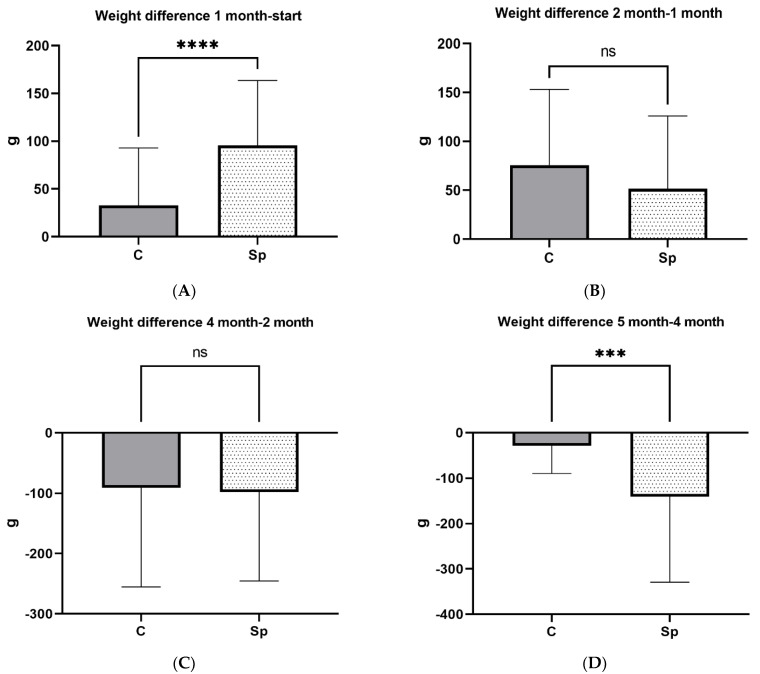
Weight differences of female mink for Control (C) and Spirulina (Sp) groups for the following intervals: between weight at 1st month of the experimental period (1 month) and weight at the start (start) (panel (**A**)); between weight at 2nd month of the experimental period (2 month) and weight at 1st month (1 month) (panel (**B**)); between weight at 4th month of the experimental period (4 month) and weight at 2nd month (2 month) (panel (**C**)); between weight at 5th month of the experimental period (5 month) and weight at 4th month (4 month) (panel (**D**)). ****: *p* < 0.0001; ***: *p* < 0.001; ns: non-significant differences.

During the first month of treatment, weight gain was higher in Sp group compared to C (*p* < 0.001). Specifically, Sp group presented an increase of 93.72 g in comparison to C group, which was 40.36 g (Figure 2). Average weight of Sp group was 1696.92 g, whereas weight of C group was 1624.36 g. A tendency (*p* = 0.067) for a higher weight was detected in animals of Spirulina group during the second month of the experiment in comparison with C group. Females in Sp group remained numerically heavier until the onset of lactation on month 4 of the experiment. Subsequently, they lost more weight than those in control group. During the last month of the experiment Sp group lost 140.40 g, compared to C group which lost 28.64 g (*p* < 0.001). No statistically important difference was observed in final body weights of both groups.

Regarding weight of kits at weaning, no difference was found between two groups (*p* > 0.05) (Table 4). Moreover, weights both of male (*p* > 0.05) and female (*p* > 0.05) offspring were similar between groups.

## 4. Discussion

In the present study, the effects of dietary inclusion of *Spirulina platensis* on reproductive characteristics of female mink were investigated. In general, a mink’s diet consists of fish and fish by-products, which are rich in long chain polyunsaturated fatty acids prone to oxidative procedures [1]. Due to increased production of reactive oxygen species (ROS) in placenta, reproductive problems, such as infertility, may appear [9]. For this reason, mink industry antioxidants have been used during the reproduction period. It has been shown that dietary inclusion of carotenoids in mink diets decreased the amount of barren females and mortality of kits, while it increased number of whelped females [1]. Elsewhere, dietary supplementation of vitamin E decreased pre-weaning mortality, improved kits weight at weaning, and enhanced antioxidative status of mink dams [9]. Additionally, similar beneficial effects on reproductive parameters in mink was shown for chelated selenium [24]. In this context, we hypothesized that dietary inclusion of Spirulina, which has documented antioxidative properties, could be beneficial for the reproductive performance of female mink. Spirulina is a microalga, widely used as a nutritional supplement or natural additive for both human and animal consumption, as it presents anti-inflammatory, neuroprotective, anticancer, hepatoprotective, and antidiabetic activities. Natural constituents of Spirulina, and especially C-phycocyanin and carotenoids demonstrate beneficial effects on reproductive performance of animals [1,14,25].

For the purposes of the experiment, we have used a single level of supplementation compared to a control diet. Spirulina dose range in animals is wide and fluctuates between 10 mg/kg [26] and even 3 g/kg [27]. To our knowledge, there is only one published study in mink, in which three different concentrations (50, 200, 400 mg/animal) of Spirulina were used [16]. In our study a dose of 100 mg of Spirulina/kg of body weight was used, to investigate if similar positive effects as Berestov [21] could be achieved. It should also be noted that the level used in the current study could be affordable, based on the economic viability of the farm. Due to practical constrains it was not possible to evaluate an additional dosage.

Spirulina supplementation imposes significant effects on reproductive performance by participating in hormonal regulation, related to reproductive system, increasing fertility, restoring antioxidant status of the ovary and, also, by participating in ovary signaling [28,29,30]. According to Berestov [21], Spirulina supplementation at the level of 200 and 400 mg/animal in female mink decreased the percentage of abortive females and, at the same time, increased litter size. This is also supported by previous studies on nanny goats and doe rabbits, where Spirulina supplementation improved litter size [28,31]. Our results, regarding barren females are in line with these studies, as a tendency for a higher whelping rate was detected in animals treated with Spirulina. Regarding litter size, no difference was observed between the two groups. Although Sp group weaned more kits compared to C group, no significant statistical difference was observed. We could hypothesize that the lack of significant effect on litter size compared to Berestov [21], could be due to the lower dose of Spirulina that was used in our study. However, as it was previously described, due to practical constraints, it was not possible to include an additional dose-treatment in our study.

During the winter period, mink are exposed to a restricted diet to reach an ideal Body Condition Score (BCS). Restricted diet is followed by 3–5 days ad libitum feeding before the beginning of the mating period [32]. Flushing has been shown to positively affect the reproductive results in female mink [5]. During the first month (pre-mating period) of dietary inclusion of Spirulina in female mink a high anabolic effect was detected. This was evidenced by three times more weight gain for the Sp group compared to the C group. Meanwhile, a tendency for a higher body weight was detected in the second month of supplementation with Spirulina. In poultry, it has been shown that dietary supplementation of Spirulina may contribute to improved efficiency of feed utilization, absorbability of dietary vitamins, and digestion of nutrients [33,34]. Such procedures in the digestive system are important for female mink during the mating, gestation, and lactation period.

The energy demands of female mink alters markedly throughout the reproduction period and are high enough during the last third of gestation [5]. Although Sp group remained heavier compared to the C group, during gestation, parturition, and until the beginning of lactation (1st period), no significant statistical difference was observed. Demands during lactation period increase progressively and depend on litter size and body weight of kits. Usually, they are notably high and require extensive mobilization of energy reserves, which lead to loss of weight, especially for those with more than four litters [6,35]. These demands decline progressively when kits start to have access to solid feed. Weaning appears to remain the most stressful period for mink dams, indicated by highest levels of plasma cortisol, followed by a reduction in body weight [6]. In the last month of our experiment (month 5), during late lactation (2nd period) till kit weaning, the final body weight of female mink between two groups was similar. However, the Spirulina-treated group lost significantly more weight compared to the Control group. It could be hypothesized due to the weight difference between treatment groups before whelping, mink of the Sp group were able to mobilize more available body reserves. This higher mobilization of body reserves in the Sp group may not be regarded as a negative effect. This could be supported by the fact that litter size was similar between groups and mortality for females and their kits was also similar. Thus far, the effect of Spirulina supplementation on growth and body weight has been studied in many animal species with contradictory results. Offspring development is influenced by many factors connected to mothers’ prenatal status, including environmental, nutritional and metabolic factors [11]. Herein, kit weight at weaning did not demonstrate any difference between two groups, which is in agreement with previous studies in broiler chickens [36,37]. In contrast, Hassanein et al. [38] reported a beneficial effect on final body weight in growing rabbits when fed with Spirulina. Similar results were presented also by Kaoud [34], in which an improvement of broiler body weight following supplementation with Spirulina was observed. Khalifa et al. [28] reported that Spirulina supplementation on nanny goats from pre-breeding until the pre-natal period, as well as post-natal supplementation on their kits, improved live body weight gain to both dams and offspring.

Overall, according to our and other published results in various animal species, it could be suggested that dietary inclusion of Spirulina in female mink may lead to improved reproductive performance. Yet, given the limitations of our experiment, the exact underlying mechanism of the observed effects cannot be identified. We presume that Spirulina supplementation may have resulted in an improved oxidative and metabolic status of the female mink. This field requires further investigation.

## 5. Conclusions

The results of the present study indicate that dietary Spirulina supplementation presented a tendency for an improved whelping rate. Female mink treated with Spirulina presented a higher percentage of whelping, a lower percentage of barren females and, finally, a numerically higher number of weaned kits which resulted in more pelts for sale compared to the Control group. However, further investigation is required to determine the exact mechanism by which Spirulina regulates the function of the reproductive system of mink dams, as also higher dosages should be tested.

## Figures and Tables

**Table 1:** Chemical analysis and total phenolic content of feed.

	Control	Spirulina
Moisture (g/kg)	3.1	3.6
Protein (g/kg)	43.7	43.7
Fat (g/kg)	13.8	14.3
Ash (g/kg)	9.7	8.9
Total phenolics (ng GAE/g DM ^1^)	20.5	21.4
Arginine (g/kg)	2.6	2.6
Cysteine (g/kg)	0.4	0.4
Isoleucine (g/kg)	1.5	1.4
Leucine (g/kg)	2.9	2.9
Lysine (g/kg)	2.8	2.9
Methionine (g/kg)	0.8	0.8
Threonine (g/kg)	1.6	1.6
Tryptophan (g/kg)	0.6	0.6
Valine (g/kg)	1.7	1.7
Saturated FA ^2^ (g/kg)	2.3	2.3
Polyunsaturated FA ^2^ (g/kg)	1.6	1.9
Monounsaturated FA ^2^ (g/kg)	6.0	5.8
Total energy (TE) (KJ/g)	20.737	20.881
Metabolizable energy (ME) (KJ/g)	15.194	15.311

^1^ ng GAE/g DM = ng GAE/g of dry matter; ^2^ FA= fatty acids; *n* = 6.

**Table 2:** Chemical analysis of Spirulina.

Components	g/kg
Protein	65.75
Fat	2.57
Fiber	1.82
Ash	3.88
Aspartic acid	6.47
Threonine	2.13
Serine	2.93
Glutamic acid	9.47
Proline	3.40
Glycine	2.30
Alanine	2.13
Cysteine	0.88
Valine	2.47
Methionnine	0.23
Isoleucine	2.62
Leucine	1.47
Tyrisine	2.07
Phenylalanine	3.34
Lysine	2.14
Histidine	1.27
Arginine	3.03
Tryptophan	0.62

**Table 3:** Descriptive statistics (Mean and Standard Deviation) for Litter Size (LS) of Control (N = 39) and Spirulina (N = 44) group at birth, day 10 post-partum and at weaning, as also mortality of kits at weaning.

	Control	Spirulina	
	Mean (±SD ^1^)	Mean (±SD ^1^)	*p*-Value
LS ^2^ birth	7.4 (2.17)	7.0 (1.54)	0.270
LS ^2^ d10	5.4 (2.47)	5.3 (2.61)	0.902
LS ^2^ wean	5.0 (2.42)	4.9 (2.63)	0.720
Mortality of kits	2.3(2.70)	2.1 (2.38)	0.668

^1^ SD = Sandard Deviation, ^2^ LS = Litter Size.

**Table 4:** Descriptive statistics (Mean and Standard Deviation) for weights of dams during the experimental period and of kits at weaning for Control and Spirulina groups and total.

			Control		Spirulina	
Traits (g)	Measurement	N	Mean (±SD ^1^)	N	Mean (±SD ^1^)	*p*-Value
Weight of females	Start	50	1592.2 (70.37)	50	1603.2 (69.94)	0.435
	1st month	50	1624.4 (89.29)	50	1696.9 (95.05)	<0.001
	2nd month	40	1699.7 (116.31)	40	1751.7 (120.89)	0.067
	4th month	39	1592.6 (189.15)	44	1652.6 (156.32)	0.160
	5th month	39	1560.2 (220.62)	44	1499.5 (270.25)	0.219
Weight of kits		100	1038.7 (181.62)	100	1050.0 (178.15)	0.657

^1^ SD = standard deviation.

## Data Availability

The data presented in this study are available upon request from the corresponding author.
